# Drivers and barriers to the choice of production systems among smallholder pig farmers: Evidence from Northern Uganda

**DOI:** 10.1016/j.heliyon.2024.e41554

**Published:** 2024-12-27

**Authors:** Caleb Ibukunoluwa Adewale, Elly Kurobuza Ndyomugyenyi, Basil Mugonola

**Affiliations:** a Department of Rural Development and Agribusiness, Faculty of Agriculture and Environment, Gulu University, P.O Box 166, Gulu, Uganda; b Department of Animal Production and Range Management, Faculty of Agriculture and Environment, Gulu University, P.O Box 166, Gulu, Uganda; c Department of Aquaculture and Fisheries, University of Arkansas at Pine Bluff, Pine Bluff, AR, USA

**Keywords:** Drivers, Pig production systems, Weaner to slaughter, Farrow to finish, Farmers' choice, Multinomial logistic regression

## Abstract

Pork consumption has risen significantly in many emerging nations, prompting diverse pig production systems. This study explored the drivers and barriers to the choices of pig production systems and the challenges confronting pig farmers in Northern Uganda. Data were collected from 240 pig farmers using a structured questionnaire and analyzed using multinomial logit regression. Results revealed that 38.8 % of the pig farmers practiced the farrow-to-weaner (breeding) production system. Further, years of farming experience, access to extension service, number of initial stocks, and gender significantly influenced the choice of the farrow to finish production system. Significant predictors for the weaner-to-slaughter (fattening) system were market proximity, years of farming experience, household size, number of initial stocks, and access to extension service. It is recommended that extension services be enhanced and tailored to specific production systems, with a focus on breeding management, feeding practices, and marketing strategies to better support pig farmers. Further, investments should be made in transportation infrastructure to facilitate direct farm-to-market linkages for pig farmers.

## 1 Introduction

Meat consumption is rising globally, driving a significant increase in livestock production [1]. Over the past decade, global meat production has grown considerably, with the majority of this increase occurring in developing countries [2]. Livestock production, particularly pig farming, plays a central role in these nations' agricultural sectors [3]. Pork production, in particular, has substantially contributed to the overall growth of meat production worldwide having increased by 140 % between 1960 and 2022 [4]. Pork is obtained from domesticated pigs scientifically known as *Sus scrofa.* They belong to the family *Suidae* and the order *Artiodactyla* [5]. Pig production is among the fastest-rising sectors in global livestock, a trend expected to persist in the coming years. This sector has expanded in countries shifting from ruminant to monogastric livestock production [6]. Pork consumption worldwide has grown by 77 %, climbing from 63.5 million tons in 1990 to 113 million tons in 2022. The rising demand for pork, superior feed conversion efficiency, and higher profitability are primary factors driving the increasing popularity of pig production [7].

Although Uganda is a low-income country, with 24.5 % of its population living below the national poverty line, it stands as the largest pig producer in Eastern Africa [8]. Pig production in Uganda comes third in Sub-Saharan Africa with about 12 % of pork production in the region coming only behind Nigeria and South Africa [9]. Uganda has the highest per capita pork consumption in Sub-Saharan Africa, with an average of 3.4 kg per person per year [9]. Pig production is undoubtedly one of the major rising livestock-rearing activities in the remote communities of Uganda and has become exceptionally appealing all through the country as a way of expanding revenue, food production, and job opportunities [10]. The central region of Uganda leads in pig production as it accounts for 56 % of the total pigs produced while the northern region comes the least taking about 14 % of the pigs produced in the country [11]. The sale of pigs and their by-products offers a viable opportunity for the predominantly rural population to generate income quickly [12]. Nonetheless, as in other developing countries, Uganda is facing numerous challenges in pig production, such as insufficient capital for business growth, frequent outbreaks of diseases, inadequate animal husbandry skills, limited access to credit for purchasing farm inputs, substandard feed, low levels of farmer education, lack of veterinary services and high costs of building materials [13]. These obstacles result in low productivity and limit the ability of producers to compete effectively in both local and global markets [14]. However, pig farming is becoming more prevalent due to the growing demand for pork thus leading farmers to adopt various production systems.

Pig production systems in developing countries such as Uganda encompass the different farming operations utilized to raise pigs and are categorized according to the pigs' growth stages. These systems include the farrow-to-finish system, the farrow-to-weaner (breeding) system, and the weaner-to-slaughter (fattening) system [15]. In the farrow-to-finish system, pigs are reared from birth (farrowing) through to full maturity, which typically takes 6–7 months. Upon reaching maturity, pigs weigh between 90 and 100 kg. Economically, it is ideal to start with five sows and aim at producing a mature pig ready for the market with a weight of about 90 kg in about 6 months. Beyond 7 months, the pig enterprise starts to become uneconomical [16]. Generally, the farrow-to-finish system has the greatest flexibility and potential in the long run [17]. This system is also more demanding in terms of labor and capital and requires a long-term commitment to the pig business [18].

A farrow-to-weaner (breeding) system is where pigs are raised from the farrowing stage till the piglets are weaned from the sow. In this system, a small number of sows are used to produce piglets, which are weaned after one to two months, typically reaching an average weight of 20 kg [19]. These weaners are then sold to other pig farmers who may wish to stock their farms for either fattening or breeding purposes. This system could be an ideal business but there must be adequate management practices to produce quality weaners [20]. However, for the weaners to attract a good price, there must have been an adequate selection of genetically sound boars and sows, resulting in weaners weighing about 8 kg at 40 days of age. This production system is usually practiced under intensive rearing or semi-intensive management systems due to the adequate care and attention needed for piglets to survive [15]. Few facilities, low operating capital, and reduced amount of feed are major characteristics of this system as compared to other production systems [21]. However, the biggest challenge of this system is that smallholder pig farmers may not be able to keep up with the high demands of weaners when the pig market is volatile. Farmers may have to farrow sows in groups or batches to augment the number of weaners available during periods of increased demand [22].

In the weaner-to-slaughter pig production system also known as fattening, a pig farmer purchases weaners from other pig farmers as his/her initial stock and rears them until full maturity. Most weaner-to-slaughter pig enterprises purchase weaners weighing 13–25 kg and feed them to market weight for between 4 and 5 months [23]. The mature pigs are then sold at the farm gate or in the market. This operation allows for low labor requirements and minimum overhead costs. The weaner-to-slaughter production system can afford a crop farmer the opportunity to use local feeds to finish pigs [24]. Dung from pigs in this system can serve as an important source of manure and fertilizer for farmlands. The source, health, and quality of weaners to be purchased are important factors to be considered before venturing into this enterprise [25]. Ideally, all weaners should be sourced from a single farm to mitigate the risk of potential herd health hazards [26]. According to Kithinji [15], a weaner-to-slaughter (fattening) system could prove to be economical if good weaners are reared under intensive and semi-intensive management systems. Based on these three production system options, farmers who want to venture into pig production are faced with a choice among the pig production systems and need to consider the production cost structure, capital requirements, and associated risks of each system to attain maximum profit from pig farming.

Existing literature provides valuable insights into the characteristics and barriers in pig production [[27], [28], [29], [30], [31]]. Other studies pointed out that smallholder pig farming in Sub-Saharan Africa, particularly in Uganda, is influenced by a range of socioeconomic and environmental factors, including household income, education level, access to resources, and market demand [8,13,32,33]. Socioeconomic variables, such as higher education and larger pig herds, are linked to the use of hired labor among smallholder farmers [32]. Market demand and disease management, particularly the prevalence of African Swine Fever (ASF), are critical constraints, leading to significant economic losses for farmers [13]. ASF outbreaks are associated with reduced economic output for smallholder pig farmers, exacerbating poverty [8]. Furthermore, technological innovations and government policies also shape smallholder practices, influencing production systems [33]. However, these studies did not focus on specific production systems. Although Adewale et al. [25] assessed the drivers of the technical efficiency of three pig production systems, no study has directly assessed the factors influencing the choice between different pig production systems like farrow to finish, farrow to weaner, and weaner to slaughter particularly in Uganda, leaving an important research gap in the literature. There remains a notable gap in comprehensive studies that integrate the socio-economic and institutional dimensions influencing farmers' decision-making processes regarding production systems. The choice of pig production system, a critical factor influencing efficiency, sustainability, and profitability may vary widely among farmers and could be influenced by a range of socio-economic, environmental, and technical factors. Therefore, this study sought to examine the drivers and barriers to the choice of production systems among pig farmers in Northern Uganda. Understanding these choices is essential for supporting the development of the region's pig subsector.

## 2 Materials and methods

### 2.1 Study area

**Fig. 1:**
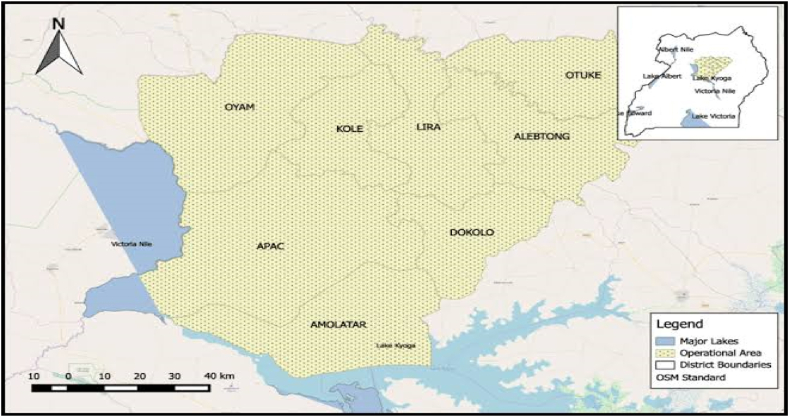
Map of the Lango Sub-Region highlighting the districts of Kole and Lira.

The study was carried out in the districts of Kole (2.3701° N, 32.7633° E) and Lira (2.2581° N, 32.8874° E) within the Lango Sub-Region of Northern Uganda (Fig. 1). This sub-region is predominantly agricultural, with most of the population depending on farming as their primary means of livelihood. The population in this sub-region is currently about 2.8 million [34]. This region, which includes districts such as Lira, Apac, Kole, and Oyam, is known for its fertile lands that support the cultivation of staple crops like millet, sorghum, and maize, as well as cash crops such as cotton and sunflower. Livestock farming, particularly cattle, pigs, and poultry, also plays a significant role in the local economy.

### 2.2 Sampling design

The study employed a cross-sectional survey, conducted between November 2021 and August 2022. Lira and Kole districts were purposefully chosen because of their high concentration of pig farmers and their geographic proximity. The reference population consisted of all pig farmers in these districts. A sampling frame was created using a list of registered farmers provided by the district agricultural officers, ensuring a comprehensive representation of the population. A stratified random sampling approach was used to ensure that farmers from the various production systems were adequately represented. The districts were first divided into three strata based on production system types, and then a total of 240 pig farmers were randomly selected from all strata in both districts, proportionally to their representation in the population. This method ensured diversity in the sample and minimized sampling bias.

### 2.3 Data collection

The survey was administered through face-to-face interviews in the field. A total of five trained interviewers were involved in data collection. The interviewers were thoroughly briefed before the survey commenced to ensure they were familiar with the study's objectives, the structure of the survey, and ethical guidelines. They were also trained on how to handle sensitive questions and ensure confidentiality. Data on the demographic of pig farmers, characteristics of pig production systems, and the challenges encountered by pig farmers in the region were gathered using a pre-tested structured questionnaire. This questionnaire featured a mix of closed and open-ended questions to ensure thorough information is obtained.

Before the main survey, a pilot study was conducted with 20 farmers from a nearby district to assess the validity of the survey instrument. Feedback from the pilot study was used to refine the survey questionnaire and interview techniques. The non-response rate was carefully monitored during data collection. Farmers who were unavailable during initial contact attempts were revisited. In cases of persistent non-response, the next available farmer from the sample frame was contacted.

### 2.4 Data analysis

#### 2.4.1 Multinomial logit regression

The logit multinomial model approach was adopted to ascertain the drivers and barriers to farmers' choices of pig production systems (Equation (1)). The Multinomial Logit Regression model is ideal for assessing the drivers behind farmers' choices of pig production systems because it accommodates multiple mutually exclusive categories (weaner to slaughter, farrow to weaner, and farrow to finish), flexibly incorporates both continuous and categorical explanatory variables, offers insights into the comparative likelihood of choosing one system over another via relative risk ratios, handles the unordered nature of these non-ranked categories, and provides interpretable coefficients that clarify the impact of different factors, ultimately making it an invaluable tool for understanding and informing decision-making and policy formulation. The model is given by;

(1) log(P(Y=Categoryk)/P(Y=Base))=β_0k+β_1kln(X1)+β_2kln(X2)+...+ε_ik

Where:

P(Y = Category k) represents the probability of choosing the k-th production system;

P(Y = Base) represents the probability of choosing the base category

log(P(Y = Category k)/P(Y = Base)) represents the log-odds of choosing the k-th production system over the base category;

β_0k is the intercept term for the k-th production system.

β_1k, β_2k, …, β_nk are the coefficients associated with each predictor (X1, X2, …, Xn) for the k-th production system.

ε_ik is the error term for the k-th choice.

X1 – X10 are outlined in Table 1.

**Table 1:** Variables used in the multinomial logit model.

Variables	Description	Expected sign
X1 = Marital status	1 = married, 0 = otherwise	+/−
X2 = Market proximity	In kilometers	+/−
X3 = Years of farming experience	Number in years	+/−
X4 = Household size	Number in years	+/−
X5 = Number of initial stocks	Number	+/−
X6 = Access to extension service	1 = yes, 0 = no	+/−
X7 = Breeds of pig	1 = large white, 0 = others	+/−
X8 = Gender	1 = male, 0 = female	+/−
X9 = Age	Number in years	+/−

## 3 Results and discussion

### 3.1 Socio-demographic characteristics of pig farmers

Results revealed that the majority (54.6 %) of the pig farmers were males (Table 2). Many of the pig farmers were married with an average age of 40 years. This could imply that pig farmers possessed the agility, youthfulness, and physical strength necessary to engage in pig-producing enterprises. They embarked on the pig farming industry intending to provide for the welfare of their families due to the high turnover of pig enterprise compared to other livestock such as cattle. These findings are consistent with Adetunji and Adeyemo [35] who noted that most pig farmers in Nigeria are married. The majority of pig farmers (75.8 %) indicated that they had ready access to markets. This may be due to the proximity of their pig farms to the marketplace. The average level of education among farmers was approximately 8 years, indicating that they possess a certain degree of literacy that allows them to effectively digest information linked to inputs and markets, resulting in improved productivity. Umeh et al. [36] corroborated this claim in their study where they reported an average of 7.71 years of education of pig farmers. Furthermore, the average number of household members for pig farmers was roughly 6. Household members might potentially serve as a source of labor for a pig production enterprise, thereby significantly reducing the expenses associated with hiring farm workers. This is in agreement with the result of Onyekuru et al. [37] which revealed a mean household size of 5 members among pig producers in Nigeria.

**Table 2:** Socioeconomic and demographic characteristics of pig farmers.

Variables	Number of respondents (N = 240)	Respondents (%)
**Gender**		
Male	131	54.6
Female	109	45.4
**Marital Status**		
Married	203	84.6
Unmarried	37	15.4
**Ready Access to Market**		
Yes	182	75.8
No	58	24.2
**Access to Credit**		
Yes	95	39.6
No	145	60.4
**Access to Extension Services**		
Yes	63	26.3
No	177	73.8
**Membership in Farming Association/Cooperative Society**		
Yes	70	29.2
No	170	70.8
	**Mean**	**Standard Deviation**
**Age**	39.6	11.672
**Years of education**	8.2	4.065
**Household size**	5.8	2.502
**Years of experience**	4.4	3.132

The average experience among farmers was roughly 4 years. A lack of experience may lead to inefficiency among pig farmers. Pig farmers with extensive experience possess a certain amount of managerial acumen, enabling them to make informed decisions that can improve output. The majority (60.4 %) of the respondents indicated a lack of access to credit. This is contrary to the findings of Fakudze and Sibandze [38] which indicated that 72 % of pig farmers had access to credit in Eswatini. However, in a study conducted by Obayelu [39], it was reported that the majority of pig farmers do not have access to credit. Access to agricultural loans is essential for increasing agricultural output and developing farming business.

In terms of access to extension services, most of the pig farmers (73.8 %) did not have access to extension services. This is contrary to the findings of Nabikyu and Kugonza [40] which stated that about 63 % of pig farmers in Uganda had access to extension services in the form of veterinary support. Extension service is crucial in the pig sector as it provides pig farmers with current information and innovative strategies to achieve optimal productivity. Experienced extension agents with sufficient expertise in pig production can certainly assist farmers in comprehending the unique characteristics of pig production systems. Furthermore, most (70.8 %) of the farmers do not belong to any farming association or cooperative society. Conversely, a study conducted by Umeh et al. [36] revealed that the majority (60 %) of pig farmers in Nigeria are members of farmers' groups. Farmer group alliances and cooperatives offer pig producers the chance to engage in bulk sales and obtain increased bargaining power.

### 3.2 Characteristics of pig production systems

Results revealed that most (38.8 %) of the pig farmers practiced the farrow-to-weaner (breeding), 30.8 % practiced the weaner-to-slaughter (fattening) and 30.4 % practiced the farrow-to-finish production system (Table 3). The most prevalent method of land acquisition among pig farmers is family land. This probably leads to a reduction in the cost of production incurred by the pig farmers, especially in terms of rent as they have access to free lands. This differs from the findings of Saka et al. [41], who found that outright purchase was the most prevalent method of land acquisition among pig farmers in Lagos, Nigeria.

**Table 3:** Characteristics of pig production systems.

Variables	Number of respondents (N = 240)	Respondents (%)
**Pig Production Systems**		
Weaner to Slaughter (Fattening)	74	30.8
Farrow to Weaner (Breeding)	93	38.8
Farrow to Finish	73	30.4
**Method of Land Acquisition**		
Freehold	2	0.8
Family land	206	85.8
Rented land	2	0.8
Purchased land	30	12.5
**Pig Breeds Farmed**		
Large white	98	40.8
Camborough	1	0.4
Large black	7	2.9
Landrace	18	7.5
Local breeds	91	37.9
Large white & Landrace	3	1.3
Camborough & local breeds	1	0.4
Large white & Local Breeds	15	6.3
Large white & Camborough	6	2.5
**Types of Feed**		
Household Wastes	1	0.4
Processed Feeds & Household Wastes	54	22.5
Local Feed Ingredients	40	16.7
Household Wastes & Local Feed Ingredients	139	57.9
All Feed Types	6	2.5
**Housing System**		
Bricks & mud	61	25.4
Timber ground	99	41.3
Tethered	59	24.6
Iron roof & cemented floor	15	6.3
Mud & wattle	6	2.5
**Water Drinking System**		
Feeding Troughs	198	82.5
Automated Nipple System	2	0.8
Improvised Bucket	40	16.7

The timber ground was the predominant type of housing in pig production, accounting for 41.3 % of the housing systems used. This could be because it is cheap to use and also easily accessible. Other closely used types of housing include bricks and mud (25.4 %) and the tethered method (24.6 %). The piglets and weaners were most likely kept in the timber ground or brick and mud housing while sows were tethered. Ndyomugyenyi and Kyasimire [42] also reported that most pig farmers used timber ground housing for their pigs in Uganda. Regarding the types of feed used for pigs, household waste and local feed ingredients were the most commonly used feed for pig production. The utilization of these feed types could be a result of their affordability and accessibility compared to processed and concentrate feeds which are very expensive. This could lead to a higher return on investment and help to reduce the costs incurred in feeding pigs. This seems to be more economical in terms of feed cost due to more local feeds being used than the more costly concentrates. Water was given to pigs mainly with the use of feeding troughs as against the automated nipple system accounting for 82.5 % indicating the lack of technological advancement in pig production in the region.

The large white and the local breeds were the most commonly reared breeds of pigs accounting for 40.8 % and 37.9 %, respectively. The predominance of the large white breed can be justified technically because of their high prolific rate and probably their disease resistance ability. They are also used to produce pigs with better characteristics through cross-breeding with local breeds. This conforms with those of Fakudze and Sibandze [38] who reported that the majority (60.7 %) of pig farmers reared large white pig breeds in Eswatini.

### 3.3 Drivers and barriers to the choice of pig production systems among pig farmers

The Prob > chi2: p < 0.000 indicates that the overall model is statistically significant (Table 4). The model with the predictor variables is significantly better than the null model with only intercept. The mean VIF (variance inflation factor) of 1.19 indicates low multicollinearity among predictors.

**Table 4:** Model estimates showing the factors influencing the choices of pig production systems among pig farmers^1^.

	Farrow to Finish	Weaner to Slaughter
	Coef. (Std. Err)	Coef. (Std. Err)
Marital status (Married)	0.942∗ (0.515)	0.706 (0.706)
Market proximity	−0.356∗∗ (0.172)	−0.422∗∗ (0.171)
Years of farming experience	0.236 (0.298)	−0.569∗ (0.340)
Household size	−0.076 (0.102)	0.823 (0.070)
Number of initial stocks	0.348∗∗ (0.165)	0.738∗∗∗ (0.168)
Access to extension service	−1.043∗∗ (0.491)	0.466 (0.418)
Pig breed (Large white)	0.648∗ (0.361)	0.564 (0.392)
Gender (Male)	−0.634∗ (0.347)	−0.288 (0.374)
Age	−0.517 (0.837)	0.492 (0.761)
Constant	0.786 (2.649)	−4.386 (2.776)
Number of obs	240	
Prob > chi2	0.0000	
Log-likelihood function	−212.36705	
Pseudo R^2^	0.2011	
Mean VIF	1.19	

*Note:* Reference category: Farrow to weaner; ∗∗∗, ∗∗ & ∗ denotes p-value at 1 %, 5 % and 10 % respectively.

The marginal effects (Table 5) revealed that years of farming experience (p < 0.1), access to extension service (p < 0.01), and gender (male) (p < 0.1) were the drivers and barriers to the choice of the farrow to finish production system over the farrow to weaner production system. Drivers and barriers to the choice of the weaner-to-slaughter production system over the farrow to weaner production system include market proximity (p < 0.1), years of farming experience (p < 0.05), household size (p < 0.1), number of initial stocks (0.01), and access to extension service (0.01). The probability of farmers choosing the weaner-to-slaughter production system over the reference category (farrow to weaner) increased with less distance to the nearest market. This may reflect the increased transportation costs, increased travel time, and potential loss of livestock quality associated with longer distances. This corroborates the findings of Jabbar and Akter [43] who reported that less distance to major markets influenced the efficiency of pig production in Vietnam. Furthermore, an increase in the number of initial stocks significantly increases the likelihood of farmers choosing the weaner-to-slaughter over the reference category. Larger stock sizes may provide economies of scale, reducing per-unit costs and potentially increasing profitability for these systems. Jabbar and Akter [43] also reported that increasing herd size influenced the efficiency of pig production in Vietnam. Pig farmers who had access to extension services were less likely to choose the farrow to finish production system possibly because these services provide better guidance for managing finishing pigs rather than breeding them. Results further revealed that access to extension services increases the likelihood of choosing weaner to slaughter production systems possibly because these services provide better guidance for managing finishing pigs rather than breeding them. According to Fakudze and Sibandze [38], access to extension services influenced the profitability of pig production in Eswatini. Okello et al. [44] also revealed that access to extension services was a significant predictor of the choice of pig health management strategies in Uganda.

**Table 5:** Marginal effects showing the factors influencing the choices of pig production systems among pig farmers.^2^.

	Farrow to Finish	Farrow to weaner	Weaner to Slaughter
	dy/dx (Std. Err)	dy/dx (Std. Err)	dy/dx (Std. Err)
Marital status (Married)	0.122 (0.086)	−0.159∗ (0.094)	0.037 (0.097)
Market proximity	−0.034 (0.028)	0.072∗∗∗ (0.026)	−0.038∗ (0.022)
Years of farming experience	0.087∗ (0.050)	0.018 (0.049)	−0.105∗∗ (0.046)
Household size	−0.021 (0.018)	0.002 (0.014)	0.018∗ (0.011)
Number of initial stocks	0.009 (0.022)	−0.096∗∗∗ (0.024)	0.087∗∗∗ (0.016)
Access to extension service	−0.230∗∗∗ (0.077)	0.079 (0.073)	0.151∗∗∗ (0.055)
Pig breed (Large white)	0.078 (0.057)	−0.115∗ (0.060)	0.037 (0.053)
Gender (Male)	−0.096∗ (0.055)	0.092∗ (0.056)	0.004 (0.050)
Age	−0.134 (0.141)	0.019 (0.128)	0.115 (0.106)

*Note:* Reference category: Farrow to weaner; ∗∗∗, ∗∗ & ∗ denotes p-value at 1 %, 5 % and 10 % respectively.

The probability of pig farmers choosing the farrow to finish production system decreased with the male gender. Cultural or social factors might influence the types of activities men engage in, or men may have different risk preferences influencing their system choice. Furthermore, pig farmers with more years of farming experience were more likely to choose the farrow-to-finish system, likely because they have the skills and resources to handle the full lifecycle of pig production. The negative and significant marginal effect indicates that with more years of farming experience, farmers are less likely to choose weaner to slaughter system compared to the reference category. Experienced farmers may prefer managing the entire production process to reduce reliance on external sources for piglets. More experienced farmers likely have the skills to manage the full lifecycle of pigs, from breeding to slaughter. In contrast, less experienced farmers may find weaner to slaughter more attractive due to the reduced complexity of breeding. YanLing et al. [45] asserted that pig production efficiency in China was increased as a result of high years of pig breeding experience. Furthermore, the positive marginal effect suggests that larger household size increases the likelihood of choosing weaner to slaughter system. Larger households may provide more labor to manage the finishing process rather than breeding pigs themselves. Larger households provide more labor to manage the growing pigs, which makes this system more feasible. This contradicts the findings of Zanu et al. [46] who found that household size negatively influenced the adoption of technology among pig farmers in Ghana.

## 4 Conclusion and policy recommendations

The current study revealed that various socio-economic factors, stock size, and accessibility to extension services influenced the choice of pig production system among farmers. These factors influence the preference for either the weaner-to-slaughter or farrow-to-finish production systems compared to the reference farrow-to-weaner system albeit positively or negatively. The significant predictors highlight areas where interventions or support could be targeted to influence system choices effectively. Based on the findings, it is recommended that:

i. Extension services should be enhanced and tailored to provide targeted advice and training for farmers based on their chosen production system. Weaner-to-slaughter systems should receive guidance on market strategies and efficient finishing techniques, while farrow-to-finish systems may benefit from breeding management and operational efficiency training.
ii. Relevant stakeholders should offer subsidies or low-interest loans to farmers to help them acquire initial stock for weaner-to-slaughter systems. Additionally, technical support should be provided for large-scale operations, particularly in health management, to ensure the sustainability of larger stock operations.
iii. Government should implement policies that improve market access for farmers, especially those in farrow to finish and weaner-to-slaughter systems, by investing in transportation infrastructure and facilitating direct farm-to-market linkages. This will help reduce transportation costs and enhance profitability for farmers closer to markets.
iv. Programs that leverage the experience of long-term farmers should be developed by offering specialized training on scaling up operations and transitioning between production systems thus helping farmers increase productivity and resilience in their operations.

## CRediT authorship contribution statement

**Caleb Ibukunoluwa Adewale:** Writing – review & editing, Writing – original draft, Visualization, Methodology, Investigation, Formal analysis, Conceptualization. **Elly Kurobuza Ndyomugyenyi:** Writing – review & editing, Writing – original draft, Methodology, Conceptualization. **Basil Mugonola:** Writing – review & editing, Writing – original draft, Methodology, Formal analysis, Conceptualization.

## Ethics statement

This study was reviewed and approved by Gulu University Research Ethics Committee (GUREC) with approval number GUREC-2022-194. Informed consent was obtained from all participants, who voluntarily signed written consent forms. The study complied with all regulations and strictly upheld confidentiality and privacy, ensuring respondents participated in safe and favorable conditions.

## Disclosure statement

The authors declare no competing interests.

## Data and code availability statement

Data will be made available on request. For requesting data, please write to the corresponding author.

## Funding

This research was funded by a grant from the 10.13039/100024788MasterCard Foundation to the Regional Universities Forum for Capacity Building in Agriculture (RUFORUM) and 10.13039/100012839Gulu University, as part of the project titled “Transforming African Agricultural Universities to meaningfully contribute to Africa's Growth and Development (TAGDev)”.

## Declaration of competing interest

The authors declare that they have no known competing financial interests or personal relationships that could have appeared to influence the work reported in this paper.

## Footnotes

1 Heteroskedasticity robust standard errors are reported.

2 Standard errors were calculated using the delta method based on the robust heteroskedasticity standard errors in Table 4.
